# Clairvoyance and the War

**Published:** 1916-11-11

**Authors:** 


### The Hospital

The Workers' Newspaper of Administrative Medicine and Institutional Life, Administration, National Insurance and Health.

No. 1588, Vol. LXI. SATURDAY, NOVEMBER 11, 1916. PRICE ONE PENNY.

### CLAIRVOYANCE AND THE WAR.

When human reason fails, being at the end of its present resources, its master the human mind turns to non-rational expedients. Who should know this better than medical men, familiar as they are with superstitious and half-superstitious essays in therapeutics on the part of patients, or of lay persons to whom patients listen? The subject of Magic, the forthcoming Fitzpatrick lectures at the Royal College of Physicians remind us, has no little medical interest appertaining to it. And at the present day its employment, indeed other than for sickness, is far from having ceased. Numbers of those devoted to a game, once overdone, but now in desuetude and seeming far away, will remember that when searching for a ball in long grass they sometimes heard themselves murmuring a sort of invocation to their invisible property. Turning from the ridiculous to the sublime, we see just now some of the countless war-afflicted seeking news of their absent or wounded ones, even of their dead. from crystal-gazers or other surviving thaumaturgists. Nor is this latter exemplification of our opening aphorism confined to uneducated persons, or, what is not the same thing, to the unintelligent either; or even to the undistinguished. A prominent physicist, who, to be sure, has all along showed sympathetic interest in clairvoyance, just lately published a book containing meditations on the nature of his separation from a slain son. An equally prominent poet, writing in a London newspaper, has encouraged the friends of the "missing" to hope for reliable information by the means and channels now discussed.

Such advocacy lends our degenerate modern magic a certain (to use a deteriorating and snobbish word) respectability. Let us try to account first for the advocacy of the scientist. The favourable prepossession mentioned is of course the first thing to note, a prepossession very naturally strengthened by fell circumstance. The antagonism between reason and feeling one recognises, but none the less every-day experience shows plentifully that the scientific mind may be rich in the common forms of deeper human emotion. Such emotion when wounded demands relief other than the slow one of time. And how that relief comes, or is apt to come, is seen when we couple together two famous sayings. Death, like the sun—wrote the great Frenchman—cannot be looked at directly. Belief in immortality has been erroneously described as a mask on the face of death. Obviously the ministrations of the clairvoyant help to strengthen that belief, and by at least so much to assuage the manifold terrors of mortality.

The case of the poet, as might be expected (for those who liken scientific imagination to artistic imagination understand neither) is different. Here the favouring prepossession, instead of being an important factor only, is almost the whole of the cause. We are not inclined, as did an anything but anti-clerical medical contemporary, to speak seriously to M. Maeterlinck about his "rubbish," to reproach him with encouraging popular credulity, with recurring to the "barbarities of mediæval thought" and to the "fetishism of primitive peoples." For how, being, as far as contemporary opinion may tell, a true poet, can he help doing so? It is a little disconcerting to think that this article of grave remonstrance would be recognised by the school of Freud as an unconscious thesis on a tenet of their master. "Such hopeless mental confusion is natural to the savage." But it is also natural to the poet. Even in this materialistic age, does not Mr. Yeats believe in fairies and in the Rosy Cross? Did not Watts-Dunton take gipsy fortune-tellers seriously, and Rossetti look for omens? But such is merely the superficies of the subject. Whoever would *approfondir* it should read an impressive and comprehensive paper dealing with the "double" or "wraith" motive in literature, which appeared a year or two back in *Imago.* Here are first brought together all cognate savage beliefs, myths, folk-lore—things like mirror superstitions, animistic ideas concerning shadows, water reflections, pictures, legends of possession, and so forth. This primitive lore in nearly all its variants and details is then shown to be given back, unconsciously yet with surprising exactness, by a group of writers of undoubted imaginative genius, a group possessing other common features, consisting as it does of such names as Poe, De Musset, Guy de Maupassant, Chamisso, Dostoievsky, and Wilde. The "William Wilson" of the American author, and the "Dorian Gray" of the English one, will be readily recalled. If, then, M. Maeterlinck, inspired by his unhappy country's misfortunes, speaks out of the promptings of his poet nature and not "as the dull brain perplexes and retards," it is no cause for wonder or censure.

The same clemency may hardly be extended to the, at best, rather dubious purveyors of this factitious consolation. Yet even they might make out a case for consideration, pointing to those higher placed and in better repute, who stand on ground which in reality is very little firmer. When in future some esoteric crank treats physical ills and fails, he will probably (seeing the issue of a recent legal trial) have to suffer for it. But when life is not at stake, and comfort of mind stands to be gained, then at a time of woe such as the present it seems unwise and unmerciful to penalise these latter-day soothsayers. It is also (dangerous subject!) quite unfair. Sufficient for now, perhaps, to rank them in that category of the ancient philosopher—namely, with professors of the (very) little arts. It is a company to which time will bring large accessions.

